# Effects of long-term metformin administration associated with high-intensity interval training on physical performance, glycogen concentration, GLUT-4 content, and NMR-based metabolomics in healthy rats

**DOI:** 10.1590/1414-431X2024e13276

**Published:** 2024-08-23

**Authors:** V.J. Bastos-Silva, H. Spineli, J.C. Guimarães, K.S.C. Borbely, J.S. Ursulino, T.M. Aquino, E.S. Bento, P.P.M. Scariot, F.A.B. Sousa, G.G. de Araujo

**Affiliations:** 1 Laboratório de Ciências Aplicadas ao Esporte, Instituto de Educação Física e Esporte, Universidade Federal de Alagoas, Maceió, AL, Brasil; 2 Grupo de Pesquisa Aplicada ao Desempenho e Saúde, Centro Universitário CESMAC, Maceió, AL, Brasil; 3 Laboratório de Biologia Celular, Instituto de Biologia e Ciências da Saúde, Universidade Federal de Alagoas, Maceió, AL, Brasil; 4 Núcleo de Análise e Pesquisa em Ressonância Magnética Nuclear, Instituto de Química e Biotecnologia, Universidade Federal de Alagoas, Maceió, AL, Brasil; 5 Laboratório de Fisiologia Aplicada ao Esporte, Faculdade de Ciências Aplicadas, Universidade Estadual de Campinas, Campinas, SP, Brasil

**Keywords:** Biguanide, Physical exercise, HIIT, Health

## Abstract

The aim was to investigate the long-term effects of metformin ingestion on high-intensity interval training on performance, glycogen concentration (GC), GLUT-4 content, and metabolomics outcomes in rats. Fifty male Wistar rats were randomly divided into baseline, metformin (500 mg daily), and control groups. Training consisted of 4 sets of 10 jumps with 30 s of passive recovery per day, 5 days/week for 8 weeks. The intensity equivalent was 50% of body mass (BM) in the first four weeks and 70% of BM in the last four weeks. The animals were submitted to a weekly jump test until exhaustion at 50% of BM. Serum and tissues were collected at baseline and after 4 and 8 weeks for biochemical and metabolomics analysis. The number of jumps increased in the Control group without a significant difference between groups at 4 and 8 weeks. GLUT4 was lower in the gastrocnemius muscle in the Metformin at the fourth week compared to Control (P=0.03) and compared to Metformin (P=0.02) and Control (P=0.01) at eight weeks. Hepatic and soleus GC were not altered by metformin. Gastrocnemius GC was lower after 8 weeks in the Metformin group compared to Control (P=0.01). Significantly lower levels of pyruvate and phenylalanine and higher levels of ethanol, formate, betaine, very low-density lipoprotein, low-density lipoprotein, and creatine were found in the Metformin compared to the Control. Although chronic administration of metformin decreased food intake and negatively influenced the synthesis of muscle glycogen, it did not significantly change physical performance compared to the Control.

## Introduction

Metformin, an oral antihyperglycemic agent, is a drug widely used in the treatment of type 2 diabetes (1). Recently, acute or short-term metformin administration has also been associated to improved physical performance in healthy organisms. In this sense, Learsi et al. (2) found that acute ingestion of metformin (500 mg) 60 min before the experimental test improved the performance in a cycle ergometer exercise at an intensity of 110% of V̇O_2_max in healthy subjects. The authors observed that the time to exhaustion was significantly longer after metformin ingestion compared to placebo, attributing this improvement to an increased alactic anaerobic contribution. More recently, Bastos-Silva et al. (3) found that acute (1 h before) ingestion of metformin (500 mg) increased the mechanical power generated at initial times (3-9 s) during a cycle ergometer Wingate test in healthy subjects.

Furthermore, de Araujo et al. (4), using the critical power method for swimming in healthy rats, found improvement in time to exhaustion and anaerobic work capacity after short-term ingestion of metformin (250 mg, for 10 days), but there was no difference in aerobic capacity. In swimmers (10 bouts of 50 m with a 3-min interval) and cyclists (12.5 km cycle ergometer time trial in hypoxic condition), short-term ingestion of metformin appears to influence glucose metabolism, anticipating the peak of lactate concentration and glycogen synthesis, but without affecting performance (5,6). These data support the hypothesis that metformin can improve anaerobic metabolism, but its contribution may be more associated to short and high-intensity exercises and the alactic anaerobic contribution.

The effects of metformin have been attributed to the inhibition of complex 1 of the mitochondrial respiratory chain, the decline of intracellular ATP levels, the increase of intracellular levels of ADP and AMP, and consequently the activation of AMPK (7). When AMPK is activated, there is an increase in GLUT-4 translocation (8), and through this mechanism, metformin increases peripheral glucose uptake (9,10). Furthermore, due to the reduction in mitochondrial respiration, there may be an increase in the cell's ability to form phosphocreatine and rebalance the cells' ATP and ADP concentrations (11). Together, these effects could increase the alatic and lactic anaerobic metabolisms by muscle cells during high-intensity exercise.

To date, the possible ergogenic action of metformin has been evaluated either after acute administration or after the administration for a maximum of 10 days (4). It is known that adaptations resulting from training are less likely during initial phases. Therefore, a long-term experimental design is essential to better understand the physiological adaptations related to chronic metformin intake in high-intensity exercise markers (such as glycogen concentration and GLUT-4 content), as well as other possible physiological changes and physical performance. The training method known as high-intensity interval training (HIIT) has been an attractive strategy because it shows physiological and psychological benefits after a few sessions in healthy subjects. HIIT is a training protocol that alternates short periods of high-intensity exercise with short periods of passive or active resting (12). HIIT induces an improvement in the activity of the Na+/K+ pump, helping to preserve cellular excitability and force production, in addition to the repeated metabolic disturbances in the concentrations of ATP, ADP, Pi, PCr, etc., which cause an increase in the concentration and enzymatic activity of cells; finally, the activation of AMPK and calcium-calmodulin kinase signaling pathways provides a potent stimulus for a variety of adaptations that improve the metabolic profile of type 2 fibers (13). Therefore, investigating the long-term administration of metformin during high-intensity physical exercise could potentiate the effects on the anaerobic energy system.

Our hypothesis was that the chronic administration of metformin associated with HIIT improves physical performance due to a greater utilization of the anaerobic pathway through increased protein content of GLUT-4, muscle glycogen concentration, and alatic anaerobic system mobilization. Thus, our purpose was to investigate the effects of chronic metformin ingestion on GLUT-4 protein content, muscle and liver glycogen, physical performance, lactate concentration, and NMR-based metabolomics analysis during HIIT in healthy rats.

## Material and Methods

### Sample

Fifty male *Rattus norvegicus albinus* (Wistar) aged 50 days were allocated in collective cages (5 rats/cage, 350 cm^2^/animal, 18 cm high) in a room with a 12-h light-dark cycle (18:00-06:00 lights on) and a temperature of 22±2°C, with food (commercial Nuvilab feed) and water *ad libitum*. All procedures in this study followed the ethical principles of animal experimentation (NIH Publication No. 86-23, revised in 1985) and were approved by the Federal University of Alagoas Ethics Committee in the Use of Animals (74/2017).

Initially, all animals underwent an adaptation to the aquatic environment for three weeks. Subsequently, the animals were randomly divided into two groups: metformin (n=20) and control (n=20). After the adaptation period (48 h), a baseline group was euthanized (n=10). The remaining animals were euthanized forty-eight hours after the first four weeks (metformin, n=10 and control, n=10) and after eight weeks (metformin, n=10 and control, n=10) of training.

### Adaptation to the aquatic environment

The purpose of adaptation to the aquatic environment was to reduce stress without the physiological benefits of physical training. The protocol used was similar from that of de Araujo et al. (14) and consisted of water exposure for five days/week during three weeks in individual cylinders with 30 cm diameter and water temperature maintained at 31±1°C. In the first week, the rats were placed in water 20 cm deep for 5 min, in the second week the water depth was increased to 30 cm and the rats performed 10 jumps on the cylinders with a load of 30% of body mass (BM) secured by a backpack to the animal's back, and finally, in the third week, a water depth of 30 cm was maintained and the rats swam for 5 min without load.

### Training protocol

**Figure 1:**
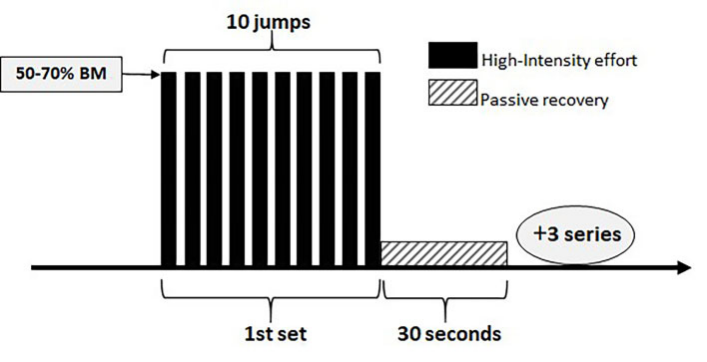
Experimental protocol. BM: body mass.

The HIIT protocol chosen followed the study by de Araujo et al. (14). The training period lasted eight weeks, with five days/week exercise sessions. The training load in the first four weeks was equivalent to 50% BM and in the last four weeks it was equivalent to 70% BM, calculated weekly to consider body weight gains. The training session consisted of four sets of 10 jumps in water (30 cm depth) with 30 s of passive recovery, out of the water (Figure 1).

### Administration of metformin

As there is still no reference value for the use of metformin in healthy organisms, we used a high dosage of metformin. The metformin group received 500 mg diluted in 1 mL of distilled water by gavage one hour before the physical exercise sessions. The animals in the control group received a solution of 1 mL of distilled water administered by gavage also one hour before exercise.

### Performance protocol

The performance test was evaluated weekly by the maximum number of jumps until exhaustion in the third training session (Wednesday) of each week. Jumps to exhaustion were performed in the last series of the session. The exhaustion criteria were determined when the rats jumped three consecutive times without reaching the water surface. The load used was maintained at 50% BM. The total load was calculated using the following calculation: [number of jumps multiplied by total mass (animal mass plus load weight)].

Before gavage and after exhaustion, blood samples (25 μL) were collected from the animals' tails at weeks four and eight for the determination of lactate concentration. Blood samples were collected using a capillary tube before and immediately, three, and five minutes after the end of the exercise to determine the resting concentration and peak lactate. Blood samples were immediately transferred to microtubes containing 25 μL of 1% fluoride and subsequently centrifuged at 1100 *g* for 10 min at 20°C for plasma separation. Plasma lactate concentrations were measured using an enzymatic method (Kit Labtest^®^, Brazil) on a spectrophotometer (UV-VIS Q798U2VS; Quimis, Brazil). The highest value was assumed to be the peak plasma lactate concentration. The difference between the peak concentration value and the resting value was considered as the delta.

### Control of food and water intake

The amount of food and water ingested per animal was measured 3 times every week, before the training or euthanasia protocol. Food intake was measured by weighing the amount of daily food left after 24 h and subtracting it from the initial amount of food. A similar procedure was adopted for measuring water intake.

### Tissue analysis

Tissues (soleus, gastrocnemius, and liver) were collected 48 h after the end of the water adaptation process (baseline) or after the last session at weeks 4 and 8 of the experimental period.

Animals were euthanized with 20% chloral hydrate (0.3 mL/100 g animal weight) for tissue excision. Tissue glycogen concentration was immediately analyzed according to the methods of Dubois et al. (15). Muscle (200-250 mg) and liver (500 mg) samples were immediately digested in 0.5 mL of 1 N KOH (30%) for 20 min and 1.0 mL, respectively. After this period, 20 µL of Na_2_SO_4_ was added for glycogen precipitation using 2.5 mL of boiling ethanol (centrifugation at 1100 *g* for 5 min at 20°C). The colorimetric assay method was analyzed using 20 µL of phenol (80%) and 2.0 mL of sulfuric acid. After 15 min of boiling, the absorbance was determined at 490 nm (15).

### GLUT-4 immunoperoxidase staining and quantification

Fresh samples were collected and immediately fixed in 4% paraformaldehyde solution (v/v) and embedded in paraffin (Sigma-Aldrich, Germany). The histological preparations were deparaffinized by means of two xylene washes followed by hydration with successive washes of ethanol in decreasing concentrations of 100, 95, and 70%, followed by a wash with distilled water and two washes with PBS. Then, immunohistochemistry assays were performed using the EXPOSE Mouse and Rabbit Specific HRP/DAB Detection IHC kit (Abcam, UK) and anti-GLUT-4 primary antibody (Abcam), both according to the manufacturer's instructions. The slides were counterstained with Mayer's hematoxylin and differentiated in running water for 10 min. The slides were then dehydrated in washes with increasing concentrations of 70, 95, and 100% ethanol, bleached with xylene, and mounted under a coverslip in Entellan medium (Sigma-Aldrich).

Microscopy was performed (Nikon DS-Ri1, Japan) and images were analyzed with DP2-BSW software (Nikon). Quantification of immunohistochemistry assays was performed using 10 images (200× magnification). Gated areas were submitted to the quantification analysis plug-in IHC Profiler from ImageJ version 1.46r public domain software (http://rsb.info.nih.gov/ij/), as described by Varghese et al. (16).

### Metabolomics analysis

For NMR analysis, blood was collected via cardiac puncture after thoracotomy into EDTA tubes (plasma), the plasma sample was centrifuged at 14,000 *g* for 15 min at 4°C, and 350 μL of serum was pipetted to an NMR tube (5 mm) with 350 μL phosphate buffer (containing trimethylsilyl propionic acid-d4 sodium salt 0.1 mM in D_2_O). NMR spectra were acquired using a Bruker 600 MHz Ascend spectrometer (Bruker BioSpin, Germany) operating at 600.13 MHz and equipped with probe PA BBO 600S3 BBF-H-D-05 Z SP. ^1^H NMR spectra were recorded using pulse sequence Carr-Purcell-Meiboom-Gill (CPMG) with water pre-saturation. For all experiments, 128 scans were recorded after 16 dummy scans, 64 k data points, spectral width of 20.0290 ppm, relaxation delay of 4 s, 2.73 s acquisition time, and 0.3 Hz line broadening. The processing spectrum was performed automatically for phase and baseline correction and calibrated to the TSP at δ 0.00 ppm, using TopSpin 3.5^®^ (Bruker BioSpin). The data matrix was prepared in MatLab^®^ software (MathWorks, version 13), where the data were superimposed, aligned, cut, and normalized. The one-dimensional spectra identified the peaks using the Human Metabolome Database (HMDB) Version 4.0 and the ChenomX NMR Suite (Chenomx Inc., Canada).

### Statistical analysis

Data are reported as means±SD. Data normality was verified using the Shapiro-Wilk test. Two-way analysis of variance (ANOVA) was used to examine the effects of drug (control *vs* metformin) and experimental time (baseline and 8 weeks) on performance, BM, and food and water intake. For glycogen concentration, GLUT-4 content, and blood lactate the effects of drug were compared (control *vs* metformin) at baseline and 4 and 8 weeks. It is important to highlight that the parameters of the test groups (metformin and control) were similar to that of the baseline (pre-training period). When a significant interaction effect was found, the Bonferroni *post hoc* test was used to identify individual differences. The level of significance was α≤0.05. Analyses were performed using the statistical software Statistica^®^ (version 10.0, StataSoft and Social Science Statistics^®^, USA). For metabolomics, PLS-DA (partial least-squares discriminant analysis) was performed to assess data's capacity to distinguish the groups from the complete set of plasma metabolites, which are presented as boxplots with median, interquartile range, and mean (baseline, metformin, and control). Finally, one-way ANOVA and Tukey *post hoc* (P<0.05) tests were performed to compare the metformin and control groups in the eighth week. For the analysis of metabolomics and parameters, we used Metaboanalyst (www.metaboanalyst.ca, Canada).

## Results

**Figure 2:**
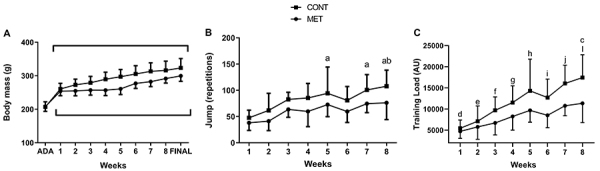
**A**, Body mass of the study groups in the eight weeks; n=20. The brackets indicate that there was a time difference in both groups in all weeks. **B**, Number of jumps in the eight weeks of training; n=20. ^a^P<0.05 compared to week 1; ^b^P<0.05 compared to week 2. **C**, Total load in the eight weeks of training; n=20. ^c^P<0.05 compared to control and metformin; ^d^P<0.05 compared to weeks 3 to 8; ^e^P<0.05 compared to weeks 4 to 8; ^f^P<0.05 compared to weeks 5, 7, and 8; ^g^P<0.05 compared to weeks 1, 2, 7, and 8; ^h^P<0.05 compared to weeks 1, 2, and 3; ^i^P<0.05 compared to weeks 1, 2, and 8; ^j^P<0.05 compared to weeks 1 to 4; ^l^P<0.05 compared to weeks 1, 2, 3, 4, and 6 (ANOVA). Data are reported as means±SD. ADA: adaptation; MET: metformin; CONT: control; AU: arbitrary units.

There was no difference in BM between the control and metformin groups during the experimental period (F=283.60; P=1.00). However, there was a time effect (F=10.90; P<0.01; Figure 2A). Regarding number of jumps, we did not observe differences between the metformin and control groups (Figure 2B). When analyzing all the eight weeks, there was a time effect only in the control group in weeks 5, 7, and 8 (F=11.06; P<0.001; Figure 2B). For total load, we found a significant effect for time (F=23.19; P<0.01) and condition (F=51.83; P<0.01) but not for their interaction (F=1.87; P=0.07; Figure 2C).

Metformin reduced food intake (F=3.86; P<0.01) in the first and second week compared to the control. Water consumption increased 11% in the first four weeks and 17.9% in the last four weeks for the metformin group compared to the control (F=20.42; P<0.01) (Table 1).

**Table 1:** Food and water intake during the 8 weeks of the study in the metformin and control groups.

Food intake (g)	Control	Metformin	P value
First week	700.72±53.54	539.92±25.23	<0.01*
Second week	709.20±25.31	558.42±37.31	<0.01*
Third week	656.32±21.83	543.25±44.24	0.27
Fourth week	665.75±19.48	557.02±70.11	0.58
Fifth week	712.75±13.64	737.75±2.33	1.00
Sixth week	696.00±25.31	736.30±40.44	1.00
Seventh week	692.35±10.53	680.80±92.63	1.00
Eighth week	699.25±16.89	649.80±133.78	1.00
Water intake (mL)	Control	Metformin	P value
First week	1023.25±70.56	1010.00±61.50	1.00
Second week	1055.00±121.90	1172.50±142.37	0.70
Third week	940.25±68.28	1093.50±183.58	0.85
Fourth week	900.00±57.79	1126.75±114.33	0.04*
Fifth week	988.00±98.99	1285.50±116.67	0.03*
Sixth week	1034.50±60.10	1229.00±120.20	0.94
Seventh week	892.50±00.71	1150.00±272.94	0.68
Eighth week	993.50±111.01	1094.50±217.08	0.99

Data are reported means±SD. 1st-4th weeks: n=4 cages, with 5 animals each, per group; 5th-8th weeks: n=2 cages, with 5 animals each, per group. *Significant difference between groups at the same time period (Student’s *t*-test).

**Figure 3:**
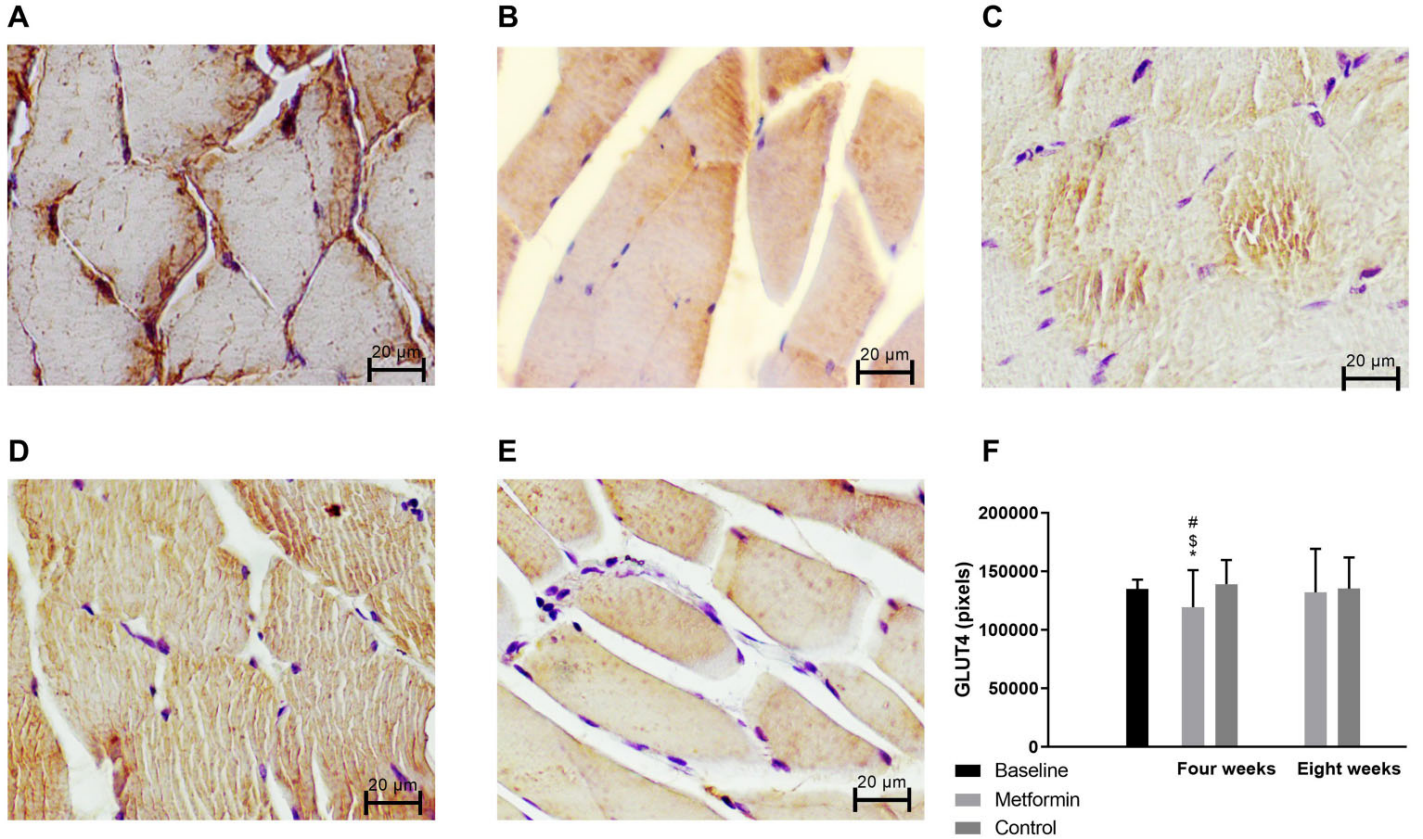
GLUT-4 expression levels in muscle tissues from the different groups. Representative immunohistochemistry images (magnification 200×, scale bar 20 μm) of gastrocnemius tissue sections with GLUT-4 staining. The sections were counterstained with hematoxylin (nucleus). (**A**) baseline; (**B**) control at four weeks; (**C**) control at eight weeks; (**D**) metformin at four weeks; and (**E**) metformin at eight weeks. **F**, Quantification of GLUT-4 protein expression in gastrocnemius tissue sections. Data are reported as means±SE. *P<0.05 compared to control at four weeks; ^#^P<0.05 compared to control at different weeks; ^$^P<0.05 compared to metformin at eight weeks (two-way ANOVA).

There was no difference between metformin and control groups at baseline (P=0.39). GLUT4 levels were lower in the gastrocnemius muscle in the Metformin group at the fourth week compared to Control (P=0.03) and compared to Metformin (P=0.02) and Control (P=0.01) at eight weeks (Figure 3).

Liver and soleus muscle glycogen content was not altered by metformin ingestion. In contrast, the glycogen content of the gastrocnemius muscle was lower in the metformin group compared to the control group at week eight (F=4.60; P=0.01; Table 2).

**Table 2:** Muscle and liver glycogen content.

	Baseline	4th week		8th week	
		Control	Metformin	Control	Metformin
Liver glycogen	1.865±1.030	2.259±1.011	2.244±0.995	2.761±0.172	2.733±0.136
Gastrocnemius glycogen	0.449±0.262	0.272±0.180	0.315±0.170	1.018±0.175^ab^*	0.561±0.346
Soleus glycogen	0.421±0.143	0.328±0.239	0.268±0.159	0.546±0.103	0.577±0.222^a^

Data are reported as means±SD (mg/100 mg). Baseline: n=10; 4-week term: n=20; 8-week term: n=20. ^a^P<0.05 compared to the 4th week. ^b^P<0.05 compared to baseline; *P<0.05 between groups in the same time period.

**Figure 4:**
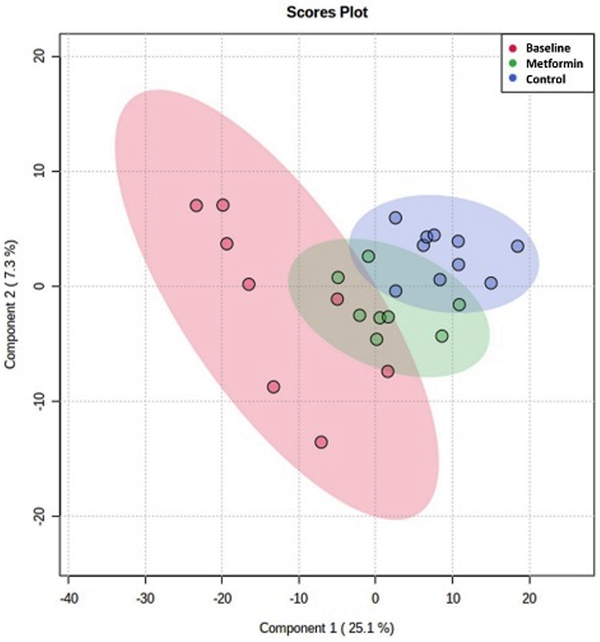
Score plot from partial least-squares discriminant analysis for baseline, control, and metformin groups.

**Figure 5:**
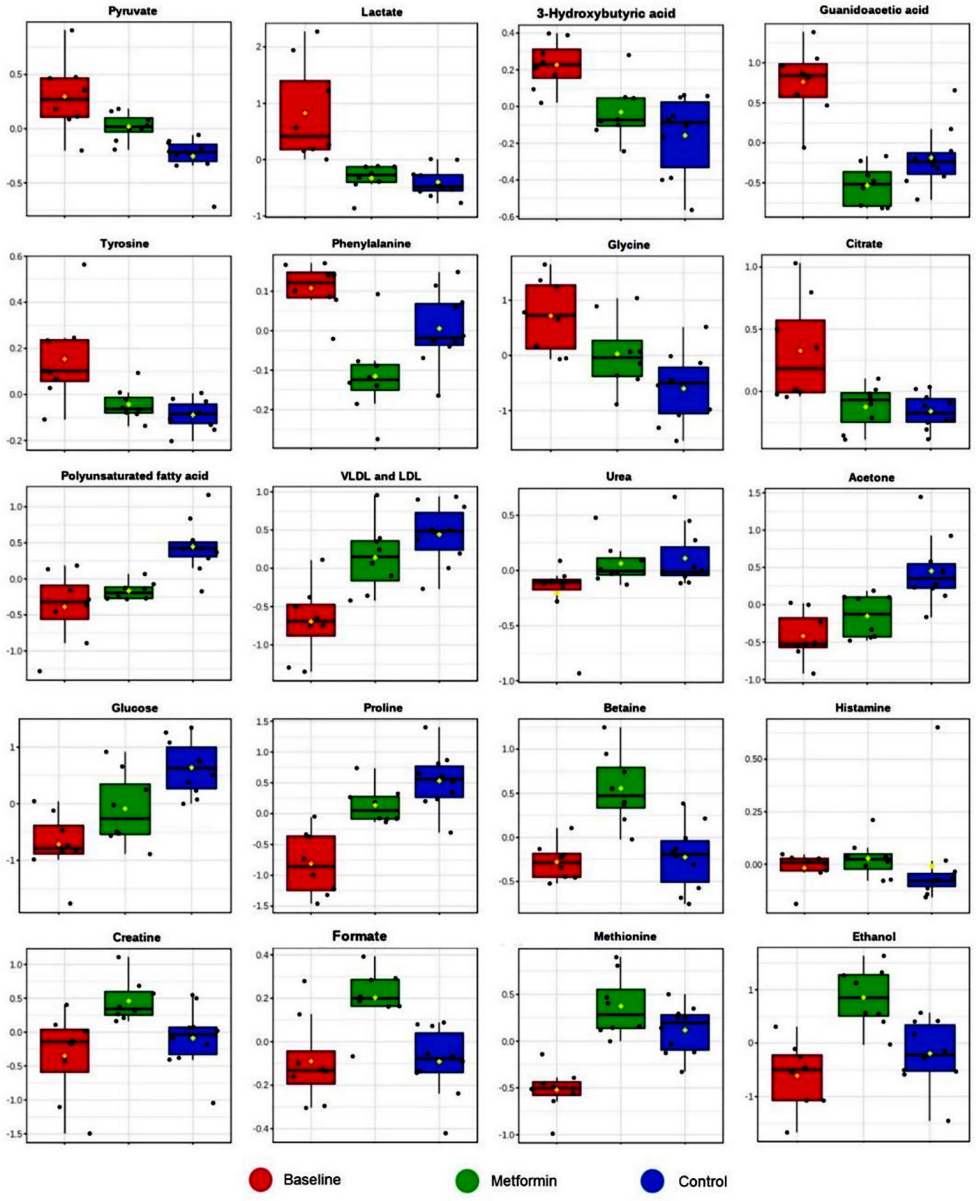
Graphic presentation of the impact of metformin and training on important metabolites in plasma. Data are reported as boxplots with median and interquartile range.

**Figure 6:**
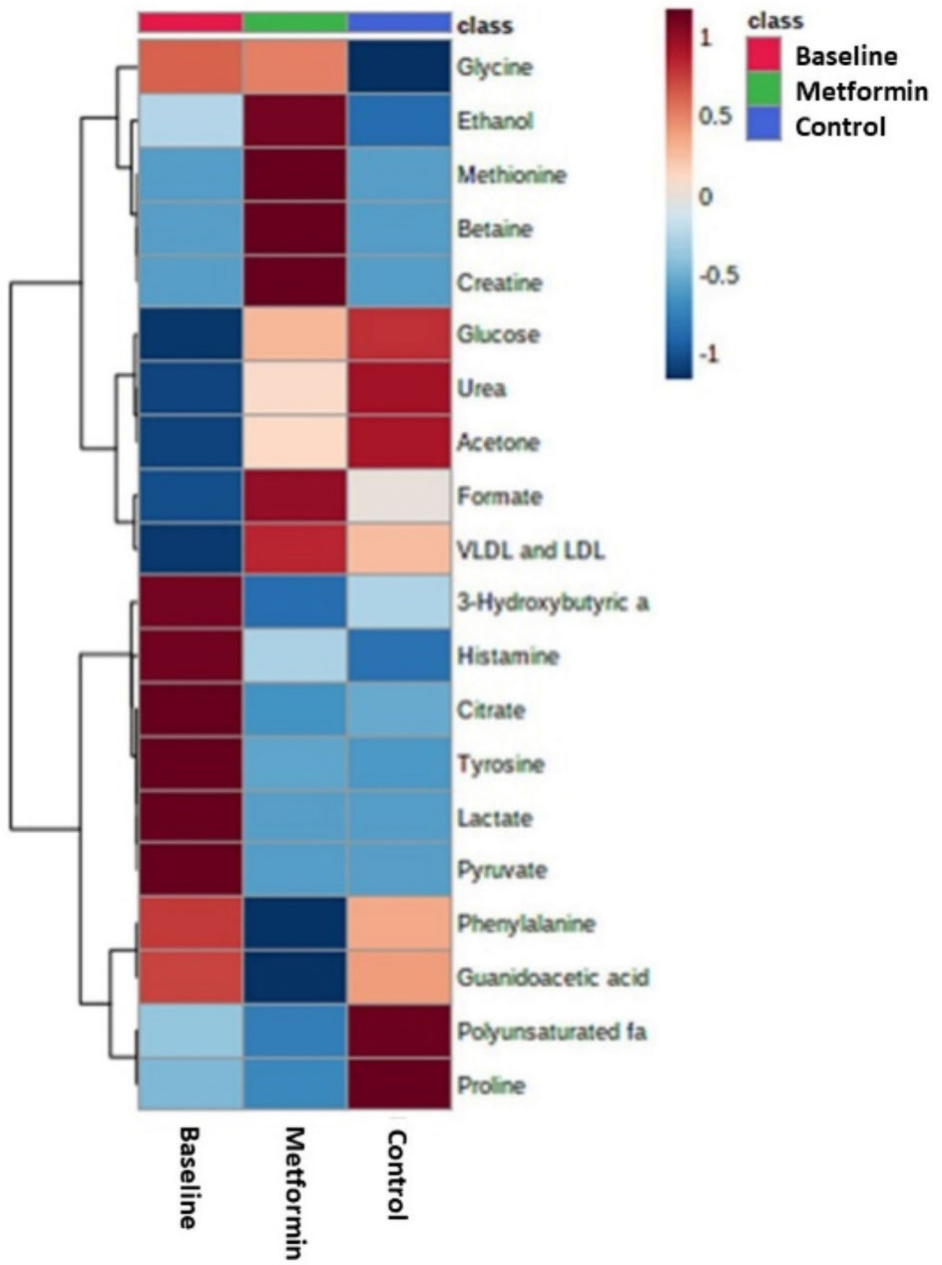
Cluster heatmap diagram for the baseline, control, and metformin groups.

PLS-DA showed a moderate discrimination and identified possible metabolic changes between the groups (Figure 4) at the end of 8 weeks. The key discriminatory features for the baseline group were the metabolites 3-hydroxybutyric acid, histamine, citrate, tyrosine, lactate, and pyruvate. On the other hand, the discriminant biomarkers for the metformin group were ethanol, methionine, betaine, creatine, formate, very low density lipoprotein (VLDL), and low density lipoprotein (LDL). Finally, glucose, urea, acetone, polyunsaturated fatty acids, and proline levels were higher in the control group. Figure 5 shows boxplots with the relative concentration of each metabolite and Figure 6 shows the cluster heatmap diagram for the groups.

There were no differences in the first four weeks and in the last four weeks in lactate concentration at rest, peak after exhaustion, and delta concentration (Table 3).

**Table 3:** Lactate concentration (mM) in the two groups at the two time points during rest and at exhaustion.

	4th week		8th week	
	Control	Metformin	Control	Metformin
Rest	1.51 ± 0.85	3.44 ± 1.09	3.64 ± 1.37	5.94 ± 2.01
Exhaustion	13.72 ± 5.83	20.88 ± 6.92	15.99 ± 4.66	18.85 ± 7.05
Delta (mM)	12.20 ± 6.13	17.44 ± 7.17	12.34 ± 4.84	12.91 ± 5.54

Data are reported as means±SD. Delta: exhaustion minus rest.

## Discussion

**Figure 7:**
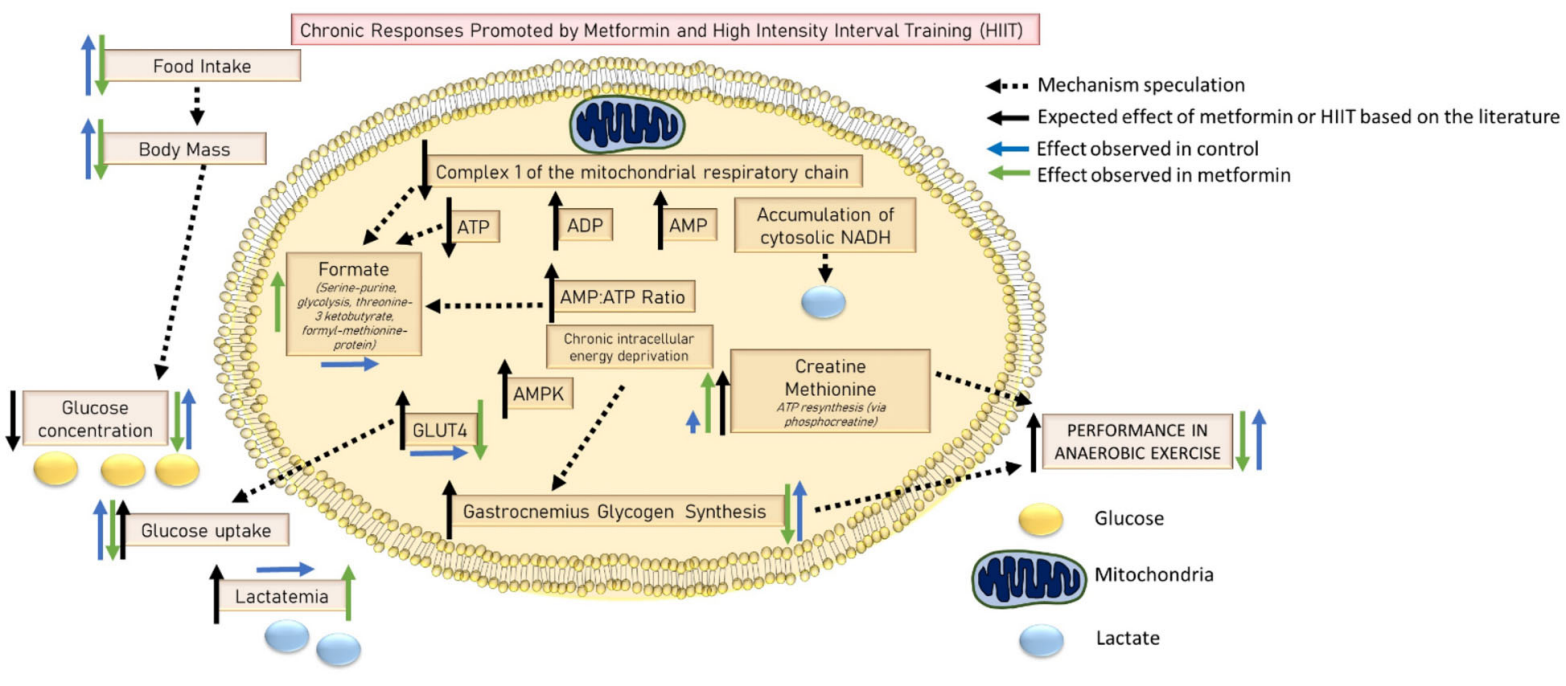
Illustration of the main findings.

This study was the first to investigate the effects of chronic administration of metformin associated with HIIT in healthy rats. Our hypothesis was rejected, as the chronic use of metformin did not increase the concentration of muscle glycogen, GLUT-4, and physical performance. On the other hand, levels of creatine, methionine, and formate (NMR findings) were higher in the metformin group than in the control, confirming its effect on the phosphagen metabolism (Figure 7).

Long-term use of metformin caused a lower food intake, as demonstrated previously 17. A possible explanation is that metformin can reduce appetite by acting on the central nervous system and attenuating the activity of hypothalamic AMP protein kinase, reducing neuropeptide Y (orexigenic) and increasing the expression of pro-opiomelanocortin (anorexigenic) 18. Metformin therapy has also been associated with improved sensitivity to leptin and insulin, and thus affects food intake 19,20. In our study, the metformin group had lower food intake compared to the control group, and the lower calorie intake may have influenced cell growth 21. Confirming this and differing from our hypothesis, a lower concentration of GLUT-4 in the metformin group was found (Figure 3). With the increased food intake in the metformin group at the end of eight weeks, we also noticed an equalization in the GLUT-4 content in this group. Cellular size and content likely develop to improve cell performance with the increase in BM and the metabolic changes that occurred 22.

Consequently, the lower food intake and GLUT-4 concentration found in the first four weeks of the metformin group may have led to a lower muscle glycogen stock at the end of eight weeks of training compared to the control group (Table 2). The literature supports that metformin increases intracellular AMP levels through compromised hepatic energy state. Therefore, it may also play an important role in controlling gluconeogenesis flow through allosteric inhibition of fructose 1,6-bisphosphatase, which is a key enzyme in gluconeogenesis 23, apparently thus inhibiting glycogen synthesis 24.

Metabolomics studies have shown that metformin ends up decreasing specific glycolytic intermediates, as well as the levels of almost all intermediates in the tricarboxylic acid cycle (Figure 5) 25. Thus, the entry of biguanides into the tricarboxylic acid cycle may decrease by the inhibition of precursors generated by carbon or nitrogen metabolism (pyruvate and glutamate, respectively) and therefore reduce the production of ATP and anabolic metabolites necessary for cell growth that are derived from the tricarboxylic acid cycle 25.

Metformin can reduce the concentrations of the intermediates of fumarate, malate, citrate, and alpha ketoglutarate, for example 26. Similarly, in our study, we found lower levels of pyruvate in the metformin group. In the absence of a functional tricarboxylic acid cycle, equivalent metabolic reductants are partially oxidized, predominantly D-lactate, succinate, formate, ethanol, and acetate. While energy in the form of ATP is generated during the excretion of acetate, energy is spent during the excretion of these other metabolic intermediates to consume reducing equivalents. In our study, the use of metformin increased the amount of ethanol and formate (Figure 5).

Our analysis revealed a decrease in VLDL and LDL cholesterol in the metformin group compared to the control 27. It is plausible that part of metformin's pleiotropic effects on lipids involves disruption of the enterohepatic circulation of bile acids, leading to increased bile acid synthesis, which eventually reduces circulating LDL cholesterol concentrations 28. In clinical practice, LDL cholesterol is one of the main markers of health risk and the need for treatment for hyperlipidemia. Lowering LDL cholesterol has been shown to reduce cardiovascular risk and mortality 27.

Regarding lactate concentration, there was no difference between the metformin and the control groups (Table 3), but the metformin presented higher absolute lactate values at rest, with lactic acidosis associated with metformin being characterized as the lactate level in the arterial blood at rest greater than 5 mM 29. In our study, the mean resting lactate was 5.94 mM in the metformin group at the eighth week and 3.64 mM in the control group, which may have negatively influenced the performance of rats in this group. This increase in lactate levels is also associated with inhibition of lactate clearance in the liver and muscles at the level of the mitochondrial respiratory chain complex, where lactate is metabolized 29. It is worth mentioning that toxicity from the use of metformin occurs after an acute deterioration in renal function 29. In our study, no metabolites related to renal function were altered using metformin.

Finally, the chronic use of metformin did not improve physical performance and impaired the improvement from physical training time (Figure 2B and C). Learsi et al. 2, de Araujo et al. 4, and Bastos-Silva et al. 3 were the first to demonstrate that metformin could exert an ergogenic effect in healthy organisms after acute or short-term of administration. One of the factors may have been the intensity used, which resulted in a high number of jumps, although it was considered high for rats, probably resulting in a large contribution of aerobic and glycolytic metabolism. In unhealthy individuals 30-33 and older adults free of chronic disease, the current literature demonstrates that the isolated strategies of aerobic exercise 34 or resistance exercise training 35 and metformin are better compared to the combination of both. This preconception was based on the idea that metformin would be a complex I inhibitor, thus impairing mitochondrial respiration and concomitantly the benefits of exercise. Acute metformin increased alactic energy contribution and high-intensity performance on Wingate in the first few seconds of testing 2,3. However, the chronic administration in healthy rats did not induce physiological responses to improve physical performance in the jump-to-exhaustion test using 50% BM of additional load.

Another factor that may have influenced the non-improvement in physical performance was the dosage used over a long period of time (500 mg for 8 weeks), approximately 6.6 times higher than the recommended therapeutic dose for diabetic animals 36. However, it is worth mentioning that this therapeutic dose is recommended for diabetic animals. de Araujo et al. 4 showed that 250 mg can improve physical performance of healthy animals, and there is still no recommendation of the ideal dose range for performance. Despite this fact, we believe that a lower dose would not have an effect different from that presented.

In clinical studies with animals or humans, doses of metformin for the treatment of pathologies (i.e., diabetes, metabolic syndrome, obesity) are well established. The use of metformin as an ergogenic for a healthy organism is new, and thus the dose used was based on a low dose commonly administered in the treatment of diabetes in humans, 500 mg. We considered the choice of a single dose as a limitation and reinforce that further studies are needed to evaluate the dose-response relationship in healthy organisms for ergogenic purposes.

Our results also suggested that HIIT improved physical performance in the control group, despite the negative interferences of confinement during the experimental period, proving to be an efficient training protocol. Studies show that housing animals in small cages leads to the animals inevitably becoming sedentary and adapting to exercise in a completely different way to animals in the natural environment 37,38. In addition, physical performance was accompanied by glycogen overcompensation in the gastrocnemius also in the control group.

On the other hand, our results showed that the soleus muscle had limited glycogen synthesis during HIIT. These results were similar to those found in the study by de Araujo et al. 14. The authors reported that in this exercise model, the gastrocnemius muscle (fast-twitch fibers) is more sensitive to glycogen synthesis than oxidative fibers due to the greater activity of glycogen synthase after exercise and the ability to replenish glycogen during physical stress.

Some limitations of the present study must be considered. The first was the test intensity, which resulted in a high contribution of aerobic metabolism due to the number of jumps. Perhaps, a high-intensity alactic test would be more appropriate to assess the effects of metformin on alactic metabolism. Another limitation was the training intensity, which was limited to 10 jumps. Exhaustive series with 10 or less maximal repetitions would increase the phosphagen system. The third limitation was the metformin dosage (500 mg, for 8 weeks), which showed no evidence of improvement in a healthy organism; further studies to test the dose-response effect on performance are needed. Finally, the lack of metabolomics analysis in different tissues (heart, skeletal muscle type I and type II, liver) limited the interpretation of the effects of metformin and physical exercise to a local level, mainly at the level of anaerobic metabolism.

In summary, our study demonstrated that chronic physical stress combined with metformin administration did not improve physical performance. In the metformin group, we also identified lower levels of glucose, pyruvate, and phenylalanine and higher levels of ethanol, formate, betaine, VLDL, LDL, and creatine compared to control. In addition, the metformin group had lower food intake, muscle glycogen overcompensation, and GLUT-4 content in healthy animals. Therefore, the chronic use of metformin in healthy organisms associated with long-term physical training does not offer any additional benefits. Further studies are needed to identify whether metformin can be used as an ergogenic in acute exercise or whether it favors prolonged protocols in other models.
